# Parametric Drug Release Optimization of Anti-Inflammatory Drugs by Gold Nanoparticles for Topically Applied Ocular Therapy

**DOI:** 10.3390/ijms232416191

**Published:** 2022-12-19

**Authors:** Gabrielle Raiche-Marcoux, Alexis Loiseau, Cloé Maranda, Audrée Poliquin, Elodie Boisselier

**Affiliations:** CHU de Quebec Research Center and Department of Ophthalmology and Otolaryngology-Head and Neck Surgery, Faculty of Medicine, Université Laval, Quebec City, QC G1V 0A6, Canada

**Keywords:** gold nanoparticles, drug release, ophthalmic therapy, anti-inflammatory drugs

## Abstract

Eye drops represent 90% of all currently used ophthalmic treatments. Only 0.02% of therapeutic molecules contained in eye drops reach the eye anterior chamber despite their high concentration. The tear film efficiently protects the cornea, reducing access to the target. Thereby, the increase in the drug bioavailability and efficiency must come from the mucoadhesion optimization of the drug delivery system. The gold nanoparticles, used as a drug delivery system in this study, already showcased ultrastable and mucoadhesive properties. The goal was to study the gold nanoparticles’ ability to release two specific ophthalmic drugs, flurbiprofen and ketorolac. The parameters of interest were those involving the loading conditions, the gold nanoparticles properties, and the release experimental conditions. The drug release was measured using an in vitro model based on dialysis bags coupled with UV–visible spectroscopy. Gold nanoparticles showed an ability to release different molecules, whether hydrophobic or hydrophilic, in passive or active drug release environments. Based on these preliminary results, gold nanoparticles could represent a promising drug delivery system for ketorolac and flurbiprofen when topically applied through eye drops.

## 1. Introduction

In North America, several millions of cataracts surgeries are carried out each year and this number will continue to grow due to the aging population [1]. There is currently no strict consensus about postoperative care but, generally, post-surgery treatment includes anti-inflammatory applications from three to eight times a day, up to four weeks for a complete treatment [2,3]. Eye drops represent about 90% of all types of ophthalmic treatments and are considered the preferred non-invasive route of drug administration [4,5]. However, this administration method is associated with low compliance from patients [6], low bioavailability of molecules, and thus very low activity [7]. Indeed, the tear film represents a challenge to be overcome for topical drug administration because of its complex structure. The tear film, located at the top on the cornea, is known to be composed of three layers: the lipid layer, the aqueous layer, and the mucoid layer [8,9]. This unique structure allows it to perform many roles, such as nutrition for the cornea, protection against micro-organisms, lubrication, and refraction of light, which has an impact on the quality of vision [10]. The tear film is also a dynamic environment; the tear turnover rate is 1–3 µL/minute for the normal eye, while the tear volume ranges from 3 µL to 6 µL [10]. Evaporation, absorption, and drainage are responsible for the dynamic balance of the tear film [11,12]. The constant renewal of the tear film is also a part of the metabolic waste products’ mechanism [13].

Different strategies are currently being developed to overcome the poor ocular bioavailability (<0.02% if the target is the anterior chamber) [14] of conventional formulation such as ocular drops. The systemic administration of drugs requires high doses in order to achieve therapeutic effects in ocular delivery which could cause potential toxicity [15]. The incorporation of permeation modifiers (including non-toxic ocular excipients) or the use of novel drug delivery systems could increase corneal absorption by enhancing the precorneal residence time [16,17]. Mucoadhesion, i.e., the ability to adhere to mucosal tissues, thus presents itself as one of the leading tactics for the topical administration of nanocarriers on the ocular surface [18]. The mucoadhesive properties of gold nanoparticles (AuNPs) have recently been demonstrated, highlighting their potential use for drug delivery systems in ophthalmology [19]. In addition to their mucoadhesive properties, AuNPs have other unique properties, making them useful for several applications in the biomedical field [20,21,22,23,24]. The polyethylene-glycol modified AuNPs developed in our laboratory are both ultrastable [19] and mucoadhesive [14], making them a strong candidate for drug delivery in ophthalmology. 

To fully benefit from the potential of these patented AuNPs (CA3043775), a market study was conducted by Zins Beauchesne and Associates, a consulting firm specialized in innovation, to fully understand how they could be the most useful. The need for anti-inflammatory drug delivery systems to increase bioavailability and efficiency was duly highlighted. Specifically, given that the estimated number of cataracts surgery is approximately 4 million per year in North America and that number will continue to grow with the aging population [25], treatment following cataract surgery is targeted. The AuNPs could help to improve the patient’s compliance by reducing the number of daily eye drop applications [26]. Furthermore, by using less therapeutic molecules, the cost per treatment could be significantly reduced in the long term [27]. Correspondingly, the anti-inflammatory drugs of choice should be eye drops currently sold on the market and currently prescribed after cataract surgery. Flurbiprofen comes as the first obvious choice; it is a cheap hydrophobic small molecule that could really benefit from the presence of an ophthalmic delivery system [2,28]. Ketorolac comes as a strong second candidate, a small molecule that is currently sold in the form of eyedrops, with the particularity of being hydrophilic [29,30,31]. In this article, flurbiprofen and ketorolac are therefore studied in drug release experiments to better understand how mucoadhesive and ultrastable AuNPs can impact their release profiles. The results from this study will also help to improve the standards of the postsurgical care of cataracts in medical communities [32,33,34]. 

This study thus aimed to determine which parameter has an impact on the release of flurbiprofen and ketorolac from our AuNP-based delivery system. The conditions used to load the drugs in the AuNPs and those used during their release were investigated. The results of this study will help to suggest optimal conditions required to maximize and control the drug release from AuNPs.

## 2. Results and Discussion

To investigate the ability of AuNPs to release the two anti-inflammatory drugs of interest, PEG_2000_-modified ultrastable AuNPs were used (the full characterization of these AuNPs can be found in a previous article [19]). To gather insights on the drug release trends of flurbiprofen and ketorolac with AuNPs, the influence of several parameters, such as the loading time or the AuNP-to-drug ratio, was assessed using dialysis bags via an in vitro method [35,36]. This is a common way of obtaining information about novel drug delivery systems as there are no universally accepted standard procedures [37]. Drug release profiles generated from dialysis-based assays are used to establish the in vivo–in vitro correlation of nanoparticle formulations, as well as to guide the formulation development and to facilitate regulatory filing and quality control [38]. It can also be used as a discriminatory tool to differentiate release behaviors, i.e., fast-release versus slow-release patterns [39]. Other methods, such as ultracentrifugation, ultrafiltration, and drug-selective electrodes, are also used to determine apparent drug release kinetics [40,41]. However, the drug release profile may be altered by external forces applied in these alternative methods. These strategies typically result in a qualitative analysis instead of the quantitative and methodological analysis from dialysis experiments. 

Herein, the release quantification of anti-inflammatory drugs (ketorolac and flurbiprofen), encapsulated into AuNPs, was performed by a colorimetric dosage using UV–visible spectroscopy [30,42]. The solution of drug-containing AuNPs was inserted in a regenerated cellulose dialysis bag with a molecular weight cut-off of 14–16 kDa. This cut-off was small enough to keep the AuNPs inside the membrane, but the pores were large enough that they were not a limiting factor for drug release in the dialysate. The release medium was nanopure water for all experiments. The calibration curves for both molecules (Figure S1) were linear, allowing the quantification experiments to be performed. 

**Figure 1:**
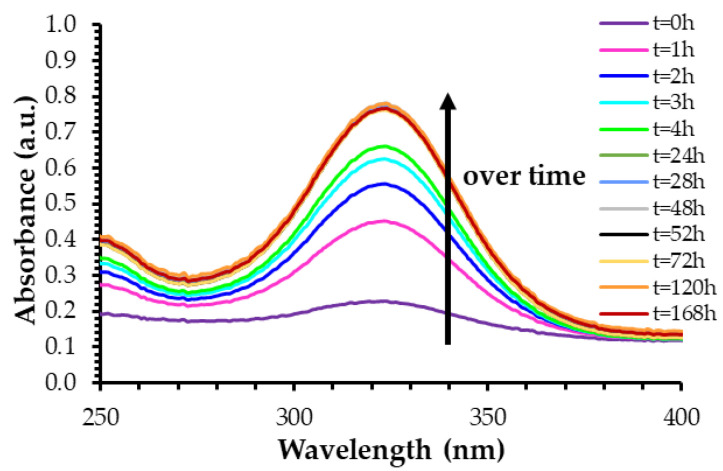
UV–visible spectra of the dialysate containing released ketorolac from AuNPs. In this example, ketorolac was loaded for three days at the AuNP-to-ketorolac molar ratio of 1:100. Spectra were taken for 7 days (equivalent to 168 h).

The drug release was measured on a 7-day period (equivalent to 168 h). During the first four hours of the drug release experiment, the absorbance intensity increased over time, indicating an increasing concentration of drug in the dialysate (Figure 1). The drug release pattern was slightly different after the fourth hour where the increase in absorbance intensity slowed down until the 28 h mark. Afterwards, and up to the 168 h mark, the UV–visible spectra of released drugs in the dialysate overlapped, meaning that the concentration of released drug in the dialysate was stable and that the drug release was complete in these conditions. As a two-step process was observed during the drug-loading experiments (see the Section 2.1.3 below), this experiment was monitored for 7 days to see if a possible second slow release would occur. However, the drug concentration in the dialysate remained stable after 28 h for both molecules at the four AuNP-to-drug molar ratios used (Figure S2). The drug release percentages and concentrations were thus calculated using the value after 28 h of experiments because all drug release patterns were quite similar, without any remarkable change through all sub-parameters.

### 2.1. Influence of Drug-Loading Parameters on Drug Release

Several parameters used during the loading of the drugs into the AuNPs can influence their release, namely the ratio of AuNPs to drug molecules, the nature of the loaded drug, and the loading time. The impact of all these sub-parameters on drug release was measured with different techniques to better assess the release profiles of the two drugs of interest from AuNPs.

#### 2.1.1. Influence of the Ratio of AuNPs to Drug Molecules on Drug Release

**Figure 2:**
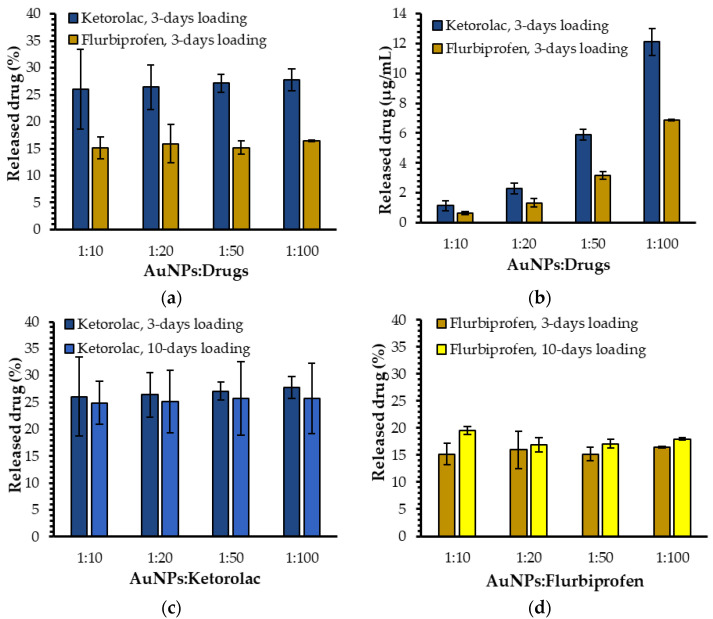
(**a**) Drug release percentages of ketorolac and flurbiprofen for the 3-day loading time; (**b**) drug release concentrations for ketorolac and flurbiprofen for the 3-day loading time; (**c**) comparison of ketorolac release percentages for the 3-day and 10-day loading times; (**d**) comparison of flurbiprofen release percentages for the 3-day and 10-day loading times.

The ratio of AuNPs to drug molecules in the loading solution corresponded to the ratio of molar concentrations of both AuNPs and drugs. For comparison purposes, the AuNP concentration remained the same throughout the experiments, while the concentration of drugs varied with the specific ratio. The chosen AuNP-to-drug-molecule ratios were 1:10, 1:20, 1:50, and 1:100. The percentages of ketorolac release were (26.0 ± 7.3)%, (26.4 ± 4.2)%, (27.1 ± 1.7)%, and (27.8 ± 2.0)% for the ratios 1:10, 1:20, 1:50, and 1:100, respectively (Figure 2a). Indeed, the concentrations of released ketorolac were (1.1 ± 0.3) µg/mL, (2.3 ± 0.4) µg/mL, (5.9 ± 0.4) µg/mL, and (12.1 ± 0.9) µg/mL (Figure 2b), meaning that the released concentration of ketorolac was dependent on the initial loading concentration, but the released concentration was always the same percentage as the initial loading concentration.

The same pattern was obtained for flurbiprofen. Indeed, the drug release percentages were (15.2 ± 2.0)%, (16.0 ± 3.5)%, (15.2 ± 1.2)%, and (16.4 ± 0.1)% for the drug-loading ratios of 1:10, 1:20, 1:50, and 1:100, respectively (Figure 2a). The drug release percentages were also very similar with all four ratios leading to released flurbiprofen concentrations of (0.6 ± 0.8 × 10^−1^) µg/mL, (1.3 ± 0.3) µg/mL, (3.2 ± 0.3) µg/mL, and (6.9 ± 0.5 × 10^−1^) µg/mL (Figure 2b). To further investigate the ability of AuNPs to encapsulate and release drugs, the encapsulation efficiency of flurbiprofen was determined, as an example and model for all samples, using an immunoprecipitation protocol with magnetic beads (see Supplementary Information for an updated protocol) [19]. The loading experiment parameters, such as the type of aqueous buffer, as well as the volumetric ratio between the drug-containing solution and the dialysate, remained the same in order to exactly replicate the same conditions used for the drug release studies. The percentages of loaded drugs depended on the initial drug concentrations. For the 1:10 ratio, (7.1 ± 0.3)% of flurbiprofen molecules were encapsulated by the AuNPs, while (21 ± 1)% of flurbiprofen molecules were loaded for the 1:20 ratio. However, the amount of loaded flurbiprofen did not have any major impact on the drug release percentages because these percentages were stable regardless of the AuNP-to-drug ratio used.

Since the percentages of the released drugs were constant, the released concentration could thus be extrapolated to other initial loading concentrations. Similarly, in the other way, the initial loading concentration of these two drugs could be selected according to the needed medical dosage.

#### 2.1.2. Influence of the Nature of the Loaded Drug on Drug Release

As previously mentioned, flurbiprofen (Ocufen from Allergan) and ketorolac (Acular from Allergan) are two anti-inflammatory drugs of similar molecular weight, currently sold as eyedrops and prescribed after cataract surgery [43]. The main physicochemical difference in interest between these two molecules is their hydrophobicity; flurbiprofen is hydrophobic whereas ketorolac is hydrophilic. The difference in drug release percentages (and implicitly in drug release concentrations) observed for ketorolac and flurbiprofen could be explained by the influence of the nature of these drugs on their ability to be released from AuNPs (Figure 2a,b). Indeed, ketorolac showed higher release percentages (about 27%) than flurbiprofen (about 16%) for all loading ratios (Figure 2a). As a hydrophilic molecule, ketorolac prefers to move from the amphiphilic environment, which the polymeric crown of AuNPs provides to the nanopure water in which the experiments take place. Conversely, as a hydrophobic molecule, flurbiprofen has more affinity for the PEGylated ligand than for the aqueous environment. This PEG-related affinity allows fewer molecules to pass into the dialysate, explaining why lower released flurbiprofen concentrations and percentages were observed in our release conditions (Figure 2a,b).

#### 2.1.3. Influence of Loading Time on Drug Release

**Figure 3:**
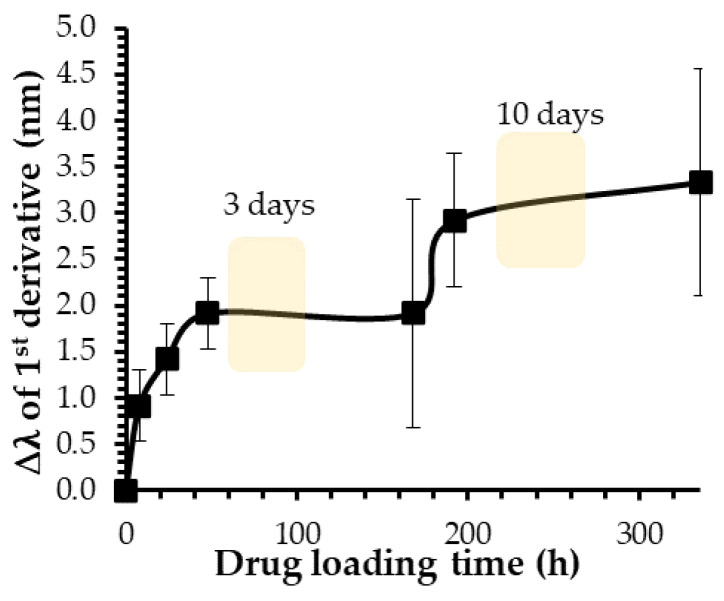
Drug-loading kinetics of ketorolac in AuNPs at the AuNP-to-ketorolac ratio of 1:100.

An interesting characteristic of AuNPs is the ability of the conduction electrons (surface plasmon) of gold atoms to coherently oscillate when irradiated by the oscillating electric field of light. The localized surface plasmon resonance (LSPR) effect causes an absorbance band in the electromagnetic spectrum, usually in the visible light range. The absorption peak can be altered by variations in different factors such as the particle size and shape, coating, the distance between particles, pH, temperature, the number and types of ligands linked to the gold core, as well as the surrounding physicochemical environment [44,45,46]. A plasmon band of AuNPs is thus sensitive to the chemical environment in which they are found. Indeed, a change in the composition in an environment close to the gold core can induce a shift in the plasmon band peak [47]. In the case of drug-loading experiments, the active molecules, which will interact with the polymeric crown of the nanoparticle, will modify the chemical environment of the gold core and cause a red shift in the plasmon band position [48]. To determine the optimal loading time for the ketorolac and flurbiprofen release experiments, the plasmonic band shift, accurately measured with the first derivative as a function of time, was used to assess the interaction between the two drugs and AuNPs [49]. UV–visible spectra were performed over two weeks (Figure 3).

During ketorolac loading experiments, the gold core was first disturbed during the first 48 h with a red shift of about 2 nm in the plasmon band wavelength (Figure 3). Then, the plasmon band position remained constant until 168 h, before being disturbed a second time, inducing a second shift of about 3 nm from the initial value. This trend was observed for both anti-inflammatory drugs studied at the four different ratios (Figure S3). The observation of this two-step gold core disturbance means that this process did not seem to depend on the drug or the drug-loading ratio. To investigate whether there was a possible correlation between this two-step process and the drug release profile, two different loading times were established for the following experiments. Indeed, two loading times of 3 and 10 days were determined, corresponding to the two plateaus observed during the drug-loading kinetics (Figure 3 and Figure S3).

Ketorolac and flurbiprofen release experiments were then performed at the four different ratios with the two loading times of 3 and 10 days (Figure 2c,d). A comparison of the drug release percentages observed in these conditions shows that the loading time did not have a significant impact on the data. Indeed, the ketorolac release percentages were quite similar for all ratios studied with values of (24.9 ± 4.0)%, (25.1 ± 5.8)%, (25.7 ± 6.7)%, and (25.8 ± 6.6)% for a 10-day loading time at the ratios 1:10, 1:20, 1:50, and 1:100, respectively (Figure 2c). Regarding the experiments with flurbiprofen, values of (19.6 ± 0.8)%, (16.9 ± 1.3)%, (17.1 ± 0.9)%, and (18.0 ± 0.3)% were observed for a 10-day loading time at the ratios ranging from 1:10 to 1:100. Despite slightly higher values observed for the 1:10 and 1:100 ratios with a 10-day loading time, these differences were not substantial enough to justify an additional 7 days of drug loading when this study was contextualized in the biomedical field, in search of an optimized pharmaceutical formulation. Considering these results, a second shift in the plasmon band peak could be induced by a reorganization of molecules in the polymeric crown stabilizing the gold core, instead of a second loading phase. Indeed, the modification of the chemical environment near the gold core could simply be explained by the displacement of the already-loaded drug molecules. Furthermore, because there was no significant difference in the drug release for flurbiprofen and ketorolac when a 3-day or a 10-day loading was chosen, all drug release results detailed in following sections were performed with this specific 3-day loading time. It is worth noting that all the experiments were still completed with a 10-day loading time and the results can be found in the Supplementary Information (Figure S4).

### 2.2. Influence of AuNPs-Related Parameters on the Drug Release 

**Figure 4:**
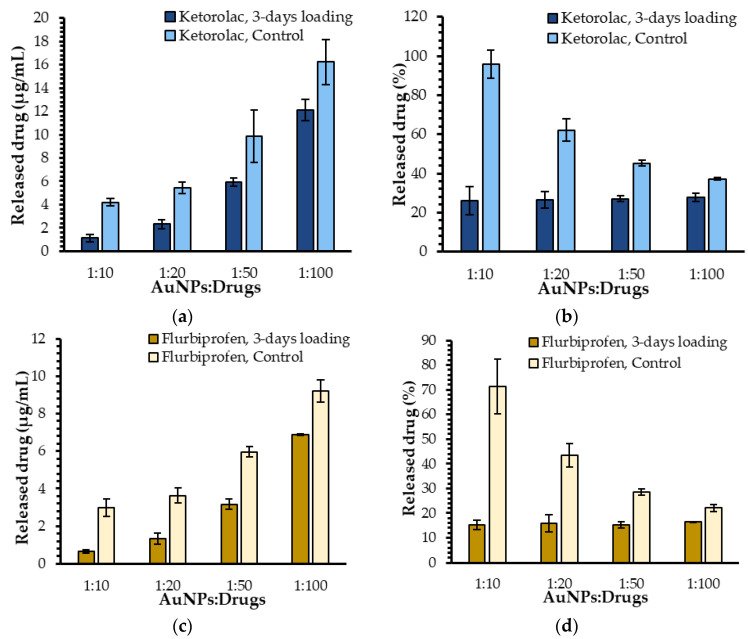
(**a**) Drug release concentrations for ketorolac with and without AuNPs; (**b**) the associated ketorolac release percentages; (**c**) drug release concentrations for flurbiprofen with and without AuNPs; (**d**) the associated flurbiprofen released percentages.

After studying the impact of different drug-related parameters on the release of ketorolac and flurbiprofen, the influence of the AuNPs themselves needs to be addressed. For this purpose, the drug release profiles of free molecules of flurbiprofen and ketorolac were compared to those observed in the presence of AuNPs. In the absence of AuNPs (ketorolac control), the release concentrations were (4.2 ± 0.3) µg/mL, (5.4 ± 0.5) µg/mL, (10 ± 2) µg/mL, and (16 ± 2) µg/mL with ratios ranging from 1:10 to 1:100 (Figure 4a). Regarding the flurbiprofen, the release concentrations were (3.0 ± 0.5) µg/mL, (3.6 ± 0.4) µg/mL, (6.0 ± 0.3) µg/mL, and (9.2 ± 0.6) µg/mL with ratios ranging from 1:10 to 1:100 (Figure 4c). In all cases, the drug concentrations in the dialysate were higher when the drugs were not encapsulated, suggesting that the AuNPs efficiently loaded both molecules and seemed to retain various amounts of ketorolac and flurbiprofen. The same could not be said when the free molecules were dispatched in the dialysate; the drug percentages in the dialysate were steadily decreasing whilst the initial drug concentration present in the dialysis bag increased. The same experiment was performed with all drugs loaded for 10 days, leading to the same trends (Figure S4).

### 2.3. Influence of Experimental Protocol Parameters on Drug Release

**Figure 5:**
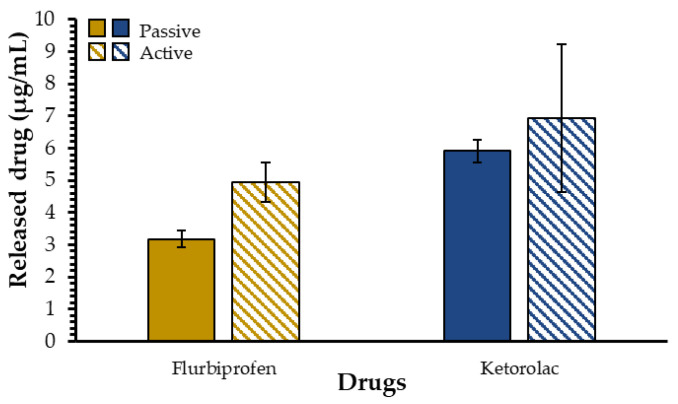
Comparison between the active and passive drug release protocols using a loading ratio of 1:50 and a 3-day loading time.

To better mimic the tear film renewal (tear turnover rate = 1–3 µL/minute) in our model, an active drug release protocol was settled on [10]. For this purpose, the dialysate was changed every 8 h for 24 h to reproduce this dynamic environment in vitro and to allow a better understanding of the impact of the tear film renewal on the ability of our mucoadhesive nanoparticles to release drugs. This modified drug release protocol was called active protocol and the results were compared to those obtained with the previously used protocol, renamed as the passive protocol for the sake of comparison. Using this active protocol, the measured concentration of released drug in the dialysate progressively increased (Figure 5). For flurbiprofen, the concentration of released drug using the passive protocol was (3.2 ± 0.3) µg/mL, while it increased to (4.9 ± 0.6) µg/mL with the active protocol. For ketorolac, the release concentration was (5.9 ± 0.4) µg/mL for the passive experiments and (7 ± 2) µg/mL for the active experiments. These results seem to indicate a trend where dialysate changes allow higher drug concentrations to be released. This could be explained by the concentration gradient and, once again, by the nature of these two anti-inflammatory drugs. Flurbiprofen, being more hydrophobic than ketorolac, has more affinity with the polymeric crown of the AuNPs than with nanopure water or ketorolac. Moreover, the differences in drug concentrations between the dialysate and the drug-containing AuNPs solution facilitate the transfer of active molecules from the AuNP solution to the dialysate. The same experiments were conducted using a 10-day loading time and the same trend was observed; the released concentration of flurbiprofen was (3.6 ± 0.2) µg/mL with the passive protocol and (4.9 ± 0.6) µg/mL with the active protocol. With ketorolac, the released drug concentration was (6 ± 2) µg/mL with the passive experiments and (7 ± 1) µg/mL under active experimental conditions (Figure S5), meaning that the 10-day loading does not affect the results obtained with the active protocol.

## 3. Materials and Methods

### 3.1. Materials

Sodium chloride (NaCl), potassium chloride (KCl), sodium phosphate dibasic (Na_2_HPO_4_), potassium phosphate monobasic (KH_2_PO_4_), and acetonitrile were all purchased from VWR International (Ville Mont-Royal, QC, Canada). Anti-polyethylene glycol antibody [PEG-B-47] was obtained from Abcam (Toronto, ON, Canada). SureBeads magnetic beads with protein A were bought from Biorad Laboratories (Mississauga, ON, Canada). Tween-20 was purchased from Fisher Scientific (Ottawa, ON, Canada). Flurbiprofen and ketorolac were obtained from Cayman Chemical Company (Ann Harbour, MI, USA). 

### 3.2. Drug-Loading Kinetics

#### 3.2.1. Preparation of AuNP Samples

Suspensions of 1 mL at AuNP-to-drug molar ratios of 1:10, 1:20, 1:50, and 1:100 were prepared using AuNPs taken from a previously published synthesis [19]. Briefly, HAuCl_4_∙3H_2_O was dissolved in an equal mix of isopropanol and acetonitrile. The PEGylated ligand, dissolved in isopropanol, was added to the gold solution. The reducing agent, NaBH_4_, was added dropwise while stirring. After stirring for 3 h, the organic solvents were evaporated using the rotative evaporator and the resulting AuNPs were suspended in nanopure water. The AuNPs were then purified by dialysis for 3 days and separated using a 0.2 µm Teflon filter. The AuNP concentration of 17.1 nmol/mL was kept constant, and the drug concentration was increased when the ratio was modified. The flurbiprofen and ketorolac samples had concentrations of 171.4 nmol/mL, 342.8 nmol/mL, 857.0 nmol/mL, and 1714.0 nmol/mL for the ratio of 1:10, 1:20, 1:50, and 1:100, respectively. Nanopure water was used to complete the volumes. Samples were shaken using a Thermomixer from Eppendorf (Germany) at 1000 rpm at room temperature for two weeks. Controls with only the drug and AuNPs were also prepared (one drug control for each ratio and one AuNP control for each replicate).

#### 3.2.2. UV–Visible Spectroscopy

UV–visible spectra were collected from 200 to 800 nm using a Cary Eclipse 50 Bio UV–vis spectrophotometer from Varian (Winnipeg, MB, Canada). A quartz cuvette (1 mm × 10 mm pathlength) from Hellma (#104.002-QS) was used (Markham, ON, Canada). UV–visible spectra were taken after 1, 2, 3, 4, 8, and 12 h and then once per day until the 14-day mark was hit. 

#### 3.2.3. First and Second Derivatives of Plasmon Band Spectra

UV–visible spectra were normalized using the intensity of the peak of the plasmon band (λ = 523 nm). Afterward, the data between 430 nm and 630 nm were derived for the first time. The inflection point of the derivative was used to determine the position of the plasmon band peak [49,50,51]. The new position of the plasmon band was subtracted from the position of the plasmon band peak of the AuNP control. The shift in the plasmon as a function of time could then be obtained. 

### 3.3. Drug-Loading Quantification

See Supplementary Information.

### 3.4. Drug Release Experiments

#### 3.4.1. Preparation of AuNPs Samples

Suspensions of 10 mL at AuNP-to-drug ratios of 1:10, 1:20, 1:50, and 1:100 were prepared. The AuNP concentration (17.1 nmol/mL) was kept constant, and the drug concentration was increased when the ratio was modified (171.4 nmol/mL, 342.8 nmol/mL, 857.0 nmol/mL, and 1714.0 nmol/mL). PBS (1X) and Tween-20 (0.0005%) were used to complete the volumes. Samples were shaken using a Multi-Tube Vortexer VWR International (Ville Mont-Royal, QC, Canada) at speed 7 at room temperature for three or ten days. Controls with only the drug and only with the AuNPs were also prepared (one drug control for each ratio and one AuNP control for each replicate).

#### 3.4.2. Parameters and Dialysis Bags

The AuNP suspensions were then moved to a cellulose dialysis bag with a 14–16 kDA molecular weight cut-off from Fisher Scientific (Ottawa, ON, Canada). This tubing was immerged in 40 mL of nanopure water in a Falcon tube. Before every UV–visible acquisition, the Falcon tube was shaken using the up-and-down technique. The maximum absorption band values for flurbiprofen (λ = 242 nm) and ketorolac (λ = 324 nm) were used for data analysis. 

## 4. Conclusions

In conclusion, this study aimed to optimize flurbiprofen and ketorolac release from AuNPs, in turn investigating several parameters. These AuNPs showed tremendous drug loading and release potential. Indeed, the AuNPs succeeded in loading and releasing hydrophobic and hydrophilic molecules. The drug release percentages were approximately 27% for ketorolac and 16% for flurbiprofen, regardless of the loading molar ratio. It was also determined that they could be used to modulate the drug dosage reaching the target, as the drug release percentages did not vary when different AuNP-to-drug ratios were used. The optimal drug-loading time seemed to be three days, following the kinetic experimental results. Waiting an additional seven days did not bring sufficient benefits to justify the extension of the loading process by a week, specifically in the biomedical and pharmaceutical context of future ophthalmic formulations. However, further testing is needed to potentially reduce this loading time. The AuNPs also demonstrated their encapsulating effect with lower drug release percentages observed in comparison with the drug alone. Further investigation into the full capabilities of the AuNPs for long-acting delivery formulations will also be needed to complement this study. Prolonged drug release kinetics could decrease the eyedrops’ application frequency for the patient, increasing their treatment compliance. Moreover, it was demonstrated that a more dynamic environment promoted an increase in drug release, which is promising because of the active nature of the tear film (Figure S6). The results from in vitro experiments, as well as their non-toxicity on human corneal epithelial cells, as previously demonstrated and published [19], showed the potential use of AuNPs as ophthalmic drug delivery systems and will be used as guidelines for further ex vivo and in vivo experiments. A better understanding of AuNP behavior in a controlled environment is crucial and will be instrumental for further experiments.

## Data Availability

Not applicable.

## Supplementary Materials

The following supporting information can be downloaded at: https://www.mdpi.com/article/10.3390/ijms232416191/s1.
