# Supplementary material for: Parametric Drug Release Optimization of Anti-Inflammatory Drugs by Gold Nanoparticles for Topically Applied Ocular Therapy
Source: Int J Mol Sci. 2022 Dec 19;23(24):16191. doi: 10.3390/ijms232416191 (PMC9786640; doi:10.3390/ijms232416191)
Supplement: Supplementary file 1 [file ijms-23-16191-s001.zip › ijms-2068364-supplementary.pdf]

SUPPLEMENTARY INFORMATION

Parametric Drug Release Optimization of Anti-Inflammatory Drugs by Gold Nanoparticles for Topically Applied Ocular Therapy

Gabrielle Raiche-Marcoux, Alexis Loiseau, Cloé Maranda, Audrée Poliquin and Elodie Boisselier *

CHU de Quebec Research Center and Department of Ophthalmology and Otolaryngology-Head and Neck Surgery, Faculty of Medicine, Université Laval, Quebec City, QC G1V 0A6, Canada

* Correspondence: elodie.boisselier@fmed.ulaval.ca

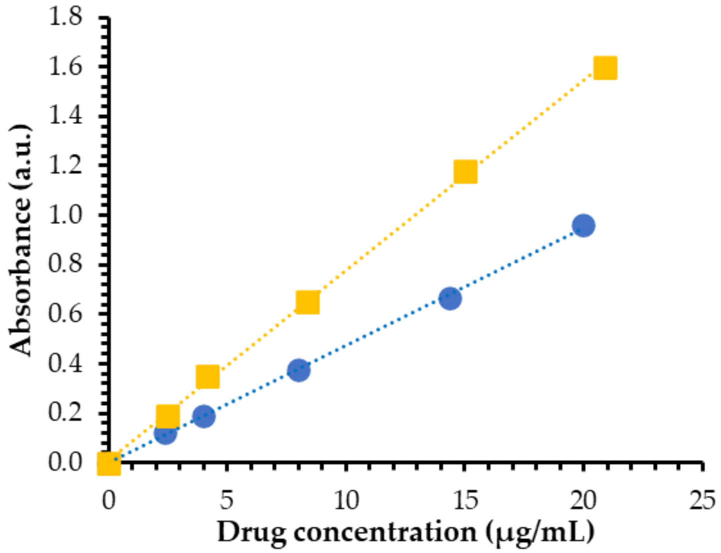

Figure S1. Calibration points and fitting lines of ketorolac (spheres in blue, absorbance at 324 nm) and flurbiprofen (squares in yellow, absorbance at 242 nm). The linear relationship between absorbance values and associated drug concentrations allows the drug quantification *via* UV-visible spectroscopy.

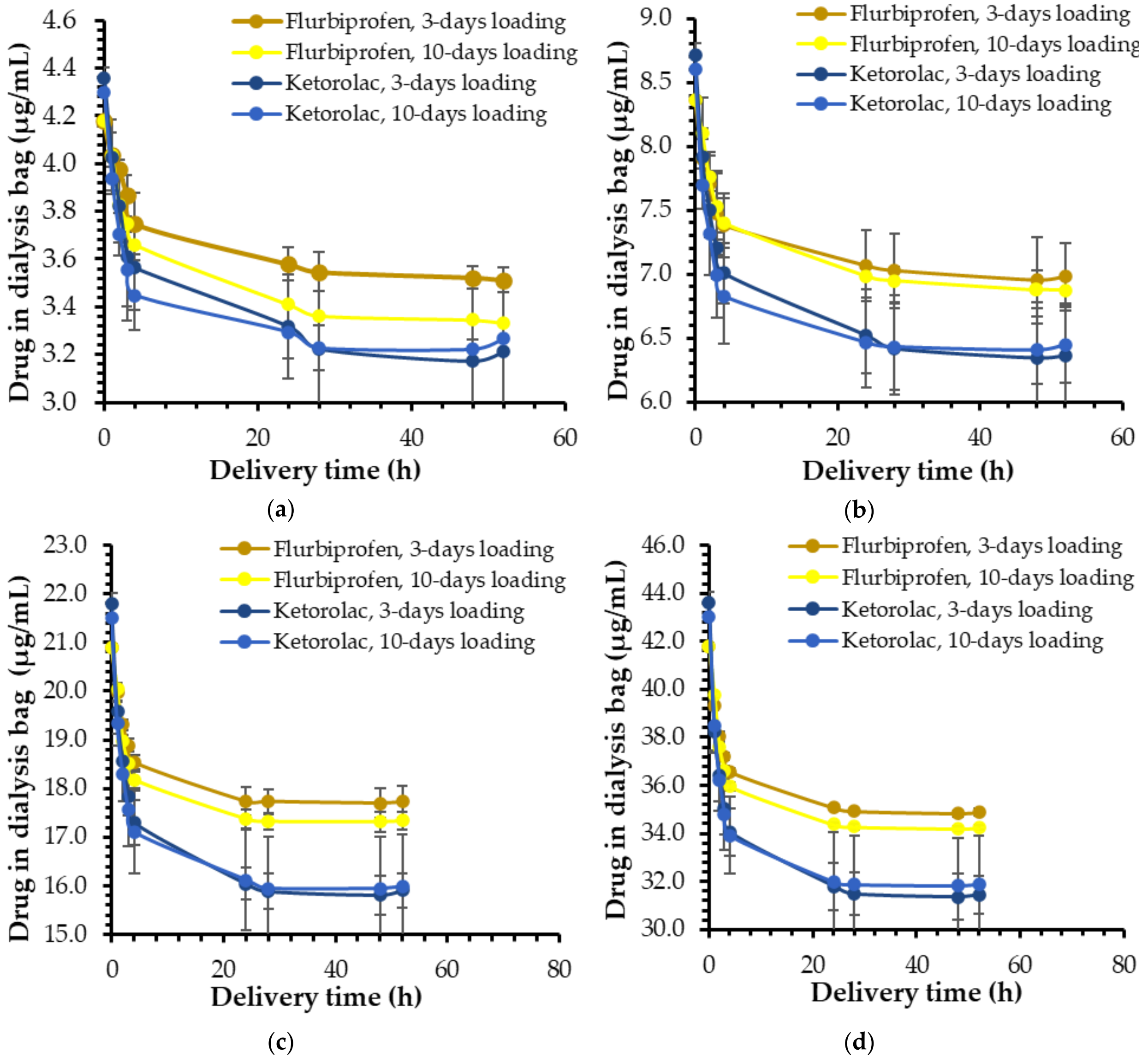

Figure S2. Drug release kinetics for ketorolac and flurbiprofen from dialysis bags. Drug loading ratios used are (**a**) 1:10, (**b**) 1:20, (**c**) 1:50 and (**d**) 1:100. For all ratios, drugs were released quickly in the first 4 hours, then released more slowly up to the 24 hours mark to finally stop after 28 hours.

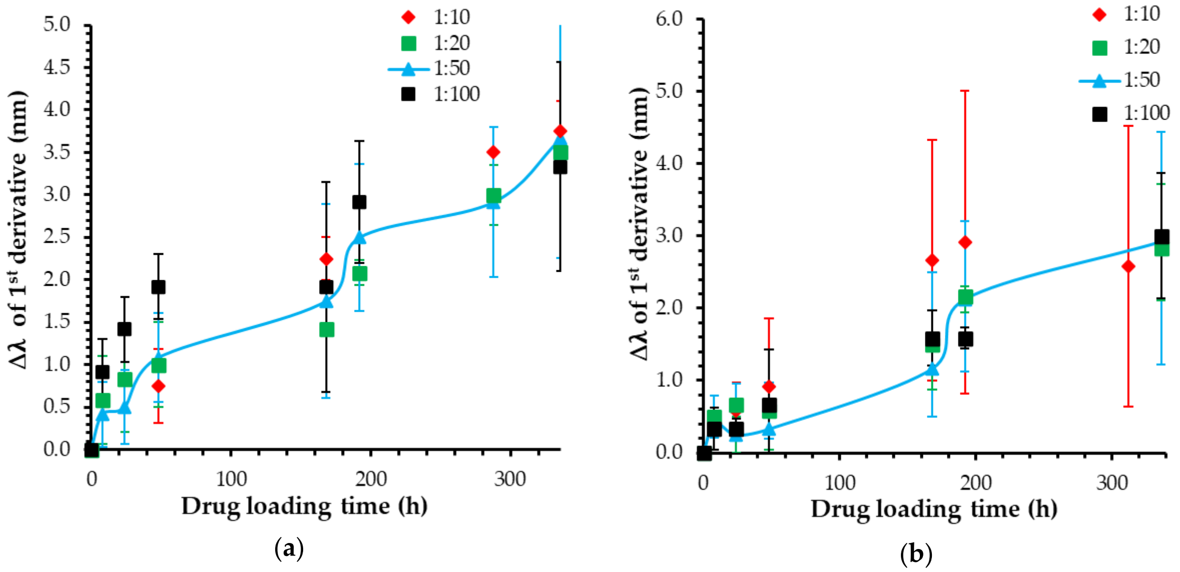

Figure S3. Drug loading kinetics using the four AuNPs:Drug ratios of (**a**) ketorolac and (**b**) flurbiprofen. Two distinct red-shifts of the plasmon band peak can be observed: the first one after 48 hours and the second one after 168 hours.

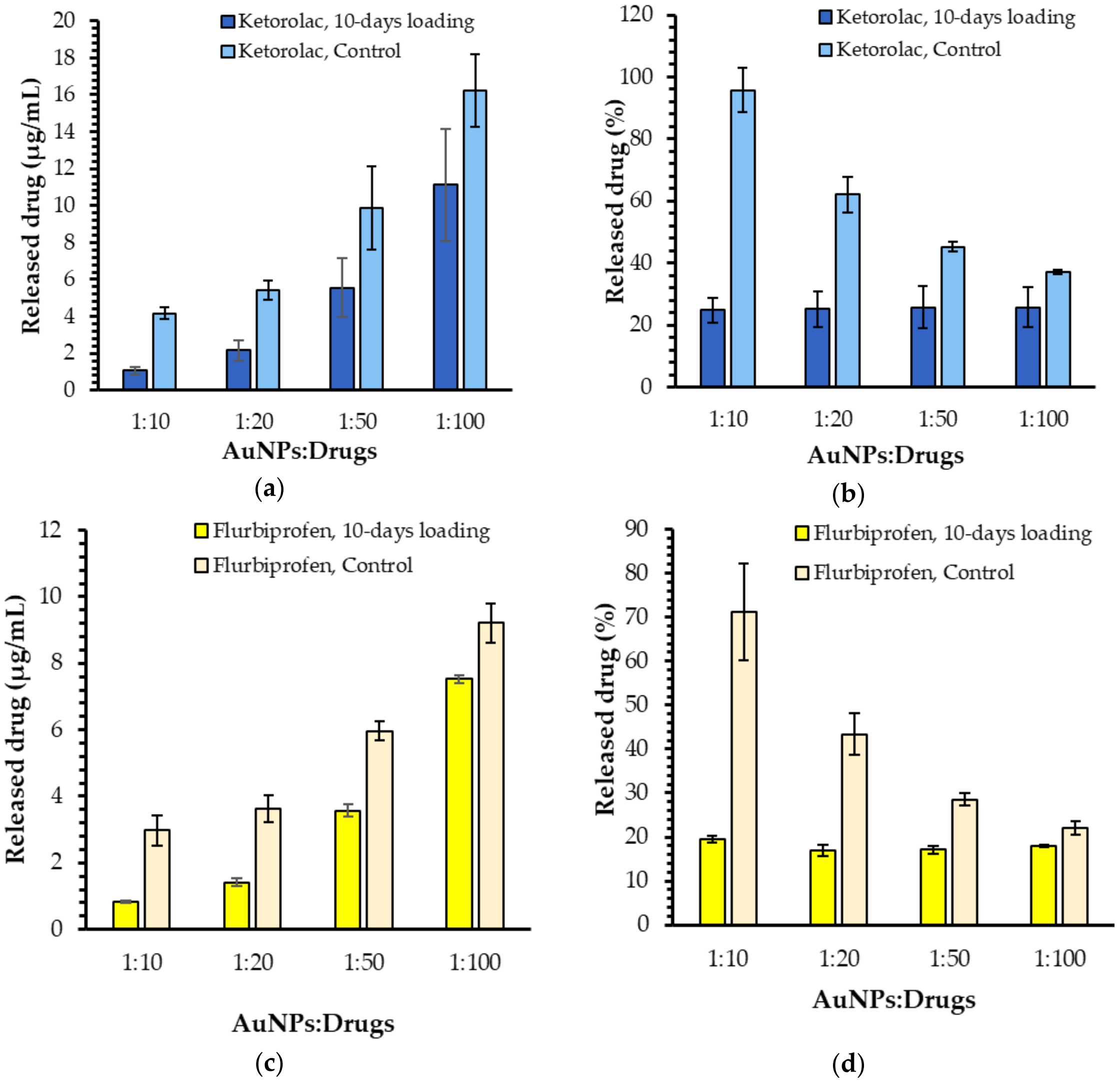

Figure S4. Drug release; (**a**) concentrations for ketorolac with and without AuNPs; (**b**) the associated ketorolac release percentages; (**c**) drug release concentrations for flurbiprofen with and without AuNPs; (**d**) the associated flurbiprofen released percentages. All drug-loading was done on a ten-days period.

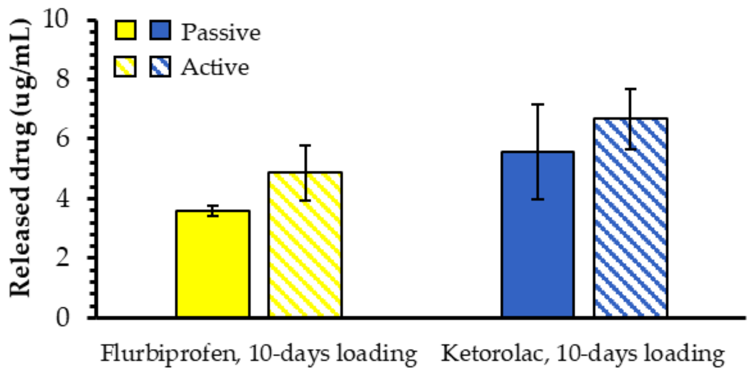

Figure S5. Comparison between active and passive drug release protocols using loading ratio of 1:50 and the 10 days-loading time.

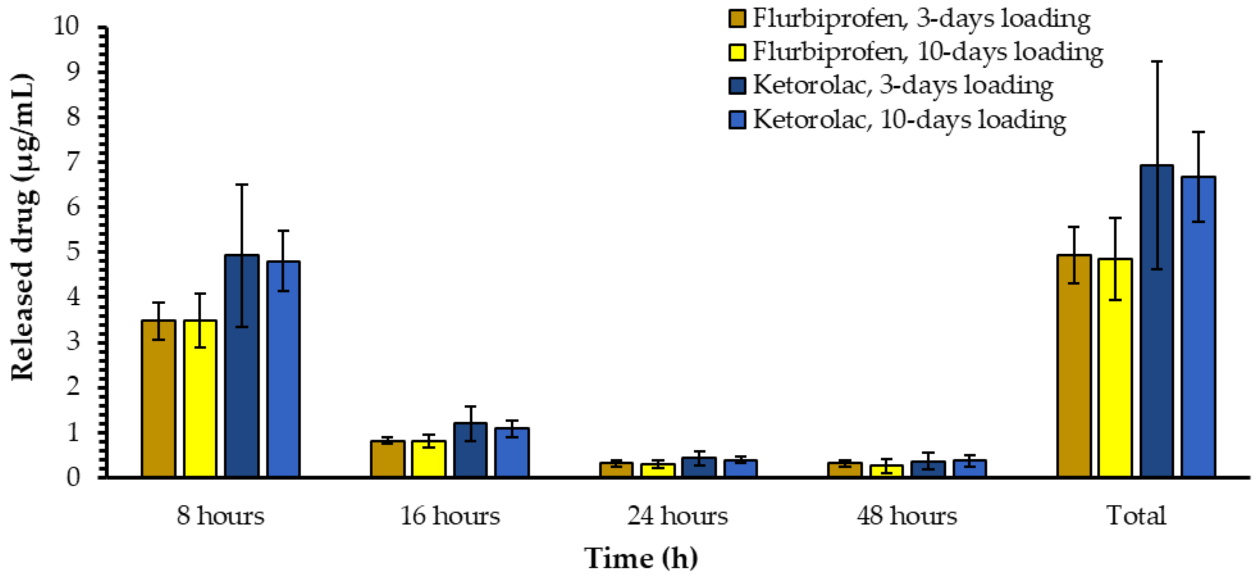

Figure S6. Concentration of flurbiprofen and ketorolac released after each dialysate change. The loading time (3 days or 10 days) does not impact the delivered drug concentration.

Drug Loading Quantification

<u>Preparation of AuNPs Samples</u>

Solutions of 500 µL at the same AuNPs:Drug ratio of 1:10 and 1:20 were prepared with the same concentrations of AuNPs (17.14 nmol/mL) and drug (171.4 and 342.8 nmol/mL). PBS (1X) and Tween-20 (0.0005%) were used to complete the volumes. Samples were shaken using a Thermomixer from Eppendorf (Germany) at 1000 rpm at room temperature for 3 days.

<u>Immunoprecipitation of the AuNPs (Adapted from SureBeads Protocol)</u>

This protocol is a slightly modified version of the previously published protocol.

A. Washing of the beads (to remove sodium azide and tert-butyldimethylsilyl ethers used as preservatives)

Add 150 µL of magnetic beads to a tube, magnetize and remove the liquid. Add 1 mL of PBS (1X) with 0.0005% of Tween-20 (PBS-T) to the beads and vortex the samples to homogeneously disperse the beads for 30 sec. Magnetize and remove the liquid. Repeat this step 2 more times to complete three washings.

B. Addition of the antibody on the magnetic beads

Add 4 g of antibody in 200 L of PBS-T and suspend the beads. Shake for 10 min at room temperature with a Thermomixer from Eppendorf (Germany) at 1200 rpm. Magnetize the beads and discard the liquid. Wash three times with 1 mL of PBS-T.

C. Addition of the sample containing the AuNPs

Add the 0.5 mL sample containing the AuNPs previously prepared. Shake overnight at room temperature with a Thermomixer at 1000 rpm. Magnetize the beads and collect the supernatant. Put it in another tube, and then re-magnetize to remove all the nanoparticles. Four magnetizations were used to ready the sample for high performance liquid chromatography (HPLC).
